# OBGYN screening for environmental exposures: A call for action

**DOI:** 10.1371/journal.pone.0195375

**Published:** 2018-05-16

**Authors:** N. M. Grindler, A. A. Allshouse, E. Jungheim, T. L. Powell, T. Jansson, A. J. Polotsky

**Affiliations:** 1 Department of OBGYN, Division of Reproductive Endocrinology and Infertility, University of Colorado, Anschutz Medical Campus, Aurora, Colorado, United States of America; 2 Department of Biostatistics and Informatics, Colorado School of Public Health, University of Colorado Anschutz Medical Campus, Aurora, Colorado, United States of America; 3 Division of Reproductive Endocrinology and Infertility, Washington University School of Medicine, St Louis, Missouri, United States of America; 4 Department of Pediatrics, Section of Neonatology, University of Colorado, Anschutz Medical Campus, Aurora, Colorado, United States of America; 5 Department of OBGYN, Division of Reproductive Sciences, University of Colorado, Anschutz Medical Campus, Aurora, Colorado, United States of America; Utah State University, UNITED STATES

## Abstract

**Background:**

Prenatal exposures have known adverse effects on maternal and neonatal outcomes. Professional societies recommend routine screening for environmental, occupational, and dietary exposures to reduce exposures and their associated sequelae.

**Objective:**

Our objective was to determine the frequency of environmental exposure screening by obstetricians and gynecologists (OBGYNs) at initial patient visits.

**Study design:**

Practicing OBGYNs were approached at the University of Colorado and by social media. The survey instrument queried demographics, environmental literacy, and screening practices. Statistical analysis was performed using Chi-square and two-sample t-test.

**Results:**

We received 312 online survey responses (response rate of 12%). Responding OBGYNs were predominantly female (96%), board-certified (78%), generalists (65%) with a mean age of 37.1 years. Fewer than half of physicians screened for the following factors: occupational exposures, environmental chemicals, air pollution, pesticide use, personal care products, household cleaners, water source, use of plastics for food storage, and lead and mercury exposure. Eighty five percent of respondents reported that they did not feel comfortable obtaining an environmental history and 58% respondents reported that they performed no regular screening of environmental exposures. A higher frequency of screening was associated with > 4 years of practice (p = 0.001), and having read the environmental committee opinion (p = <0.001).

**Conclusion:**

The majority of OBGYNs did not incorporate screening for known environmental exposures into routine practice. Reading the environmental committee opinions was strongly and significantly associated with a higher rate of screening. Improving physician comfort in counseling patients may enhance screening for exposures that affect reproductive health.

## Introduction

Women are exposed to many toxic environmental agents in their daily life and virtually every pregnant woman in the US is exposed to at least 43 different environmental chemicals [1]. Endocrine-disrupting chemicals (EDCs), which may interfere with any aspect of *in vivo* hormonal action, are of particular concern [2]. Exposure to EDCs is universal and ubiquitous, occurring through inhalation, ingestion, and contact with soil, food, and consumer products. Unlike pharmaceuticals, most environmental chemicals have entered the marketplace without comprehensive information regarding their reproductive or other long-term toxic effects [3]. We have previously reported that a specific type of plasticizer is positively associated with earlier menopause in women, implying that environmental exposures may impact reproductive lifespan [2]. Additionally, we and others have shown that exposure to particular EDCs is associated with decreased fecundity, low birth weight, preterm birth, and pregnancy loss [2, 4, 5]. Further, prenatal EDC exposure has been linked with an increased risk of childhood cancer, and genital tract and neurodevelopmental abnormalities [6].

Peri-conceptional exposure to EDC can have profound and lasting effects on reproductive health of the mother and child [7]. Indeed, evidence that links exposure of toxic environmental agents to adverse reproductive effects is sufficiently compelling that numerous governing societies promote exposure reduction [6–8]. These recommendations are summarized as follows: preventing exposure to environmental chemicals is a priority for reproductive health professionals, OBGYNS should advocate for policies to reduce exposure to toxic agents, and providers should encourage scientific investigations in environmental health as it relates to reproductive and developmental health outcomes [6–8]. These recommendations also include useful tables to help providers identify key environmental exposures as well as provide additional resources for more information [6–8]. The American College of Obstetrics and Gynecologists (ACOG) calls on their members to advocate for policies to identify and reduce exposure to environmental toxic agents while addressing the consequences of such exposure.

A previous study found that OBGYNs agreed that conducting environmental health histories would identify exposures and help prevent environmental threats [9], suggesting that OBGYNs recognize the potential harmful impact of toxic environment on peri-natal health. However, although there is consensus that there is a need to screen for environmental exposures, it is unknown to what extent these recommendations are being implemented in practice. The primary objective of this cross-sectional study was to assess the frequency of environmental exposure screening by obstetricians and gynecologists (OBGYNs) at the initial patient visit and in routine practice. Our secondary objectives were (1) to identify key areas for improvement, (2) to increase awareness to the importance of the environment on women’s health, and (3) to encourage more providers to consider screening for this exposure.

## Methods

### Sampling methodology

We conducted a cross-sectional survey of OBGYNs currently practicing medicine, representing several different sub-specialties in OBGYN and a variety of practice types. Respondents were recruited using two approaches: Department of OBGYN at University of Colorado and a social media group of physicians trained in obstetrics & gynecology. Providers at the University of Colorado were sent an email explaining the study with a link to the survey. Physicians recruited to the study via social media (Facebook physician mom group) responded to a post explaining the study with a link to the survey. All responses were anonymous and the survey was administered using REDcap. The survey was hosted at the University of Colorado Anschutz Medical Campus [10]. This study qualified for exempt status from the Colorado Multiple Institutional Review Board. Completion of the questionnaire was taken as consent. All qualified responding providers were included in main analyses, however intermittent item non-response leads to some categorical responses summing to a total less than that of the total number responding.

### Survey instrument

The 20-item survey instrument (S1 Table) collected demographics, environmental literacy, screening practices, and suggestions for future directions (S1 Table). Demographics were collected for age, specialty and/or sub-specialty, practice type (private practice, university-affiliated, other), and board eligibility/certification status. Environmental literacy was measured by asking respondents whether they routinely read the committee opinions and/or practice bulletins published by their respective professional organizations (i.e. ACOG, American Society for Reproductive Medicine, International Federation of Gynecology and Obstetrics). Provider screening practices were obtained through a question addressing screening practice patterns at the initial visit, comfort with discussing environmental exposures with patients, and general knowledge of the impact of the environment on female reproduction. We also requested suggestions on respondents’ preferences for potential interventions to improve routine screening for environmental exposures.

The use of an environmental exposure survey for new patients was assessed with the question: “Does your clinic have a routine survey administered to new patients to identify environmental exposures?” A provider’s comfort in assessing environmental history was assessed with the question: “Do you feel you have adequate training to obtain an environmental history on patients? For example, would you be able to ask the appropriate questions to exclude dangerous occupational exposures?”

### Statistical analysis

Questionnaire responses are reported as frequency and percent for categorical variables, mean and standard deviation (SD) for normally distributed continuous measures, and geometric mean and 95% confidence interval for age (a skewed continuous measure). Screening practices at new patient visits, comfort level with environmental chemical screening, and environmental literacy are reported by: provider gender, year since completion of training (<4 vs ≥4), rural vs urban/suburban practice type, residency program affiliation, current knowledge, and exposure to environmental toxic agents; with differences tested using an exact Pearson chi-square for categorical and two-sample t-tests for age. No adjustments were made for multiple comparisons in primary analysis. In sensitivity analysis, an adaptive step-up Bonferroni adjustment was applied. Analysis conducted in SAS 9.4 and graphics prepared using GraphPad Prizm 6.04.

## Results

**Fig 1:**
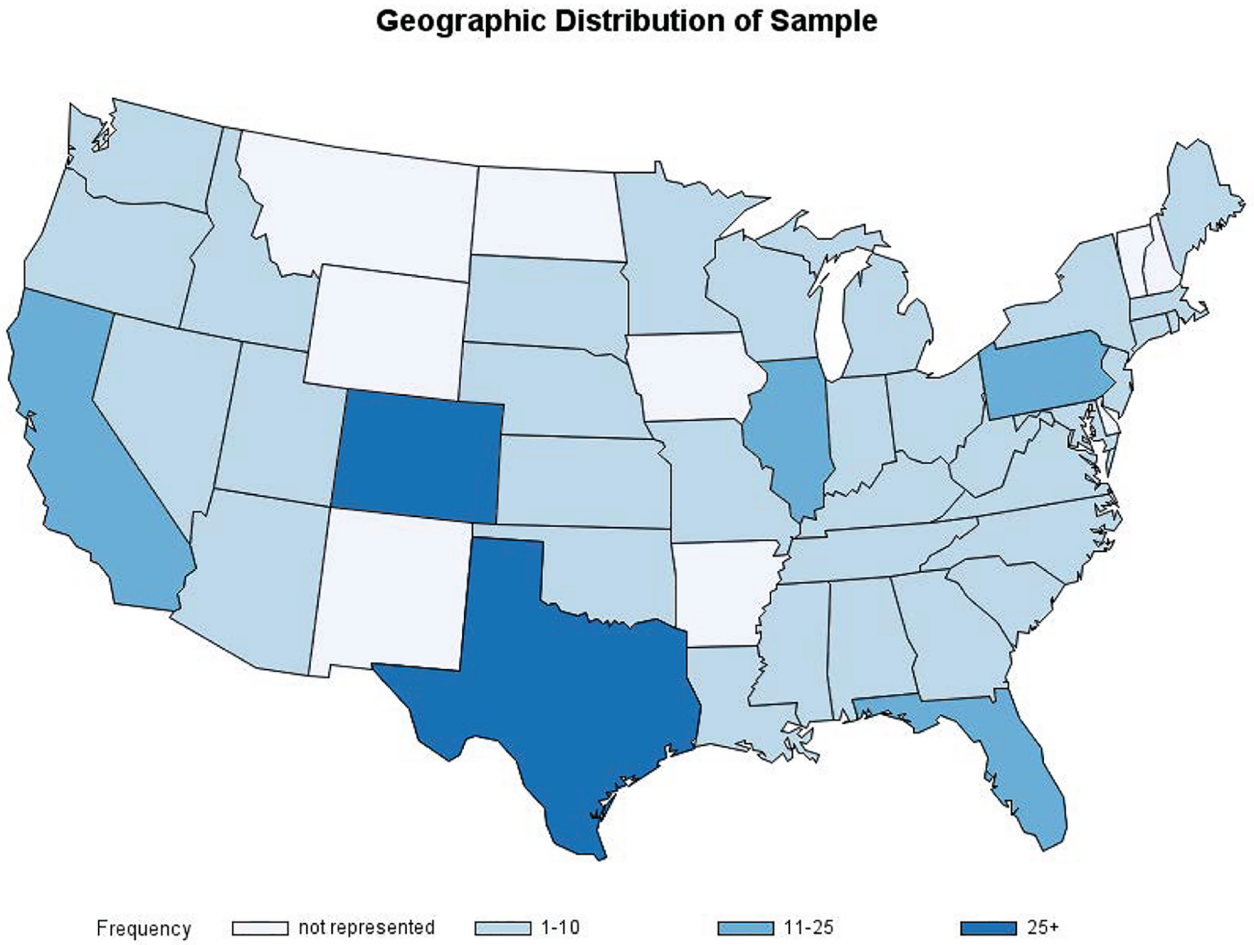
Geographic distribution of subjects.

Surveys were completed between December 29, 2016 and January 23, 2017. A total of 145 clinicians were invited via email, and 2444 physicians were members of the social media page where an invitation was posted. We received 312 survey responses, for an overall response rate of 12%. Respondents were predominantly young (mean 37.1 y), female (96%), board-certified (78%), generalists (65%) who worked at an institution affiliated with a residency program (56%), and practiced in urban locations throughout the US (Table 1). Our subjects represented regions from across the US: 18% Midwest, 13% Northeast, 28% South, 42% West (Fig 1).

**Table 1:** Basic demographics.

Characteristic	Value	All Participants n = 312 Number (% of total)
Gender	Male	13 (4)
	Female	295 (96)
Age	Geometric Mean(95%CI)	37.1 (37, 38)
Current level of training	Not Yet Board certified	69 (23)
	Board certified	238 (78)
Years since training	zero	31 (10)
	<4	83 (28)
	4–5	59 (20)
	>5–10	74 (25)
	more than 10	54 (18)
Geographic Region	Midwest	52 (18)
	Northeast	37 (13)
	South	81 (28)
	West	122 (42)
Practice location	Urban	163 (53)
	Suburban	117 (38)
	Rural	16 (5)
	Other	12 (4)
Practice Type	OBGYN	195 (64)
	Specialty	72 (24)
	Fellow/resident	37 (12)
Which best describes your practice? (Check all that apply)	General OBGYN	197 (63)
	Gynecology alone	6 (2)
	Obstetrics alone	14 (5)
	Maternal Fetal Medicine	25 (8)
	Family Planning	14 (5)
	Gynecology Oncology	7 (2)
	Minimally Invasive Surgery	9 (3)
	Urogynecology	8 (3)
	Reproductive Endocrinology & Infertility	18 (6)
	In training (fellow/resident)	37 (12)
	Other	10 (3)
What type of practice are you in?	University-based academic practice	120 (39)
	Community-based academic practice	36 (12)
	Private practice	122 (40)
	Other	30 (10)
Affiliated residency training	Yes	170 (56)

Data are number of study subjects (%) or geometric mean and 95% Confidence Interval for age.

**Fig 2:**
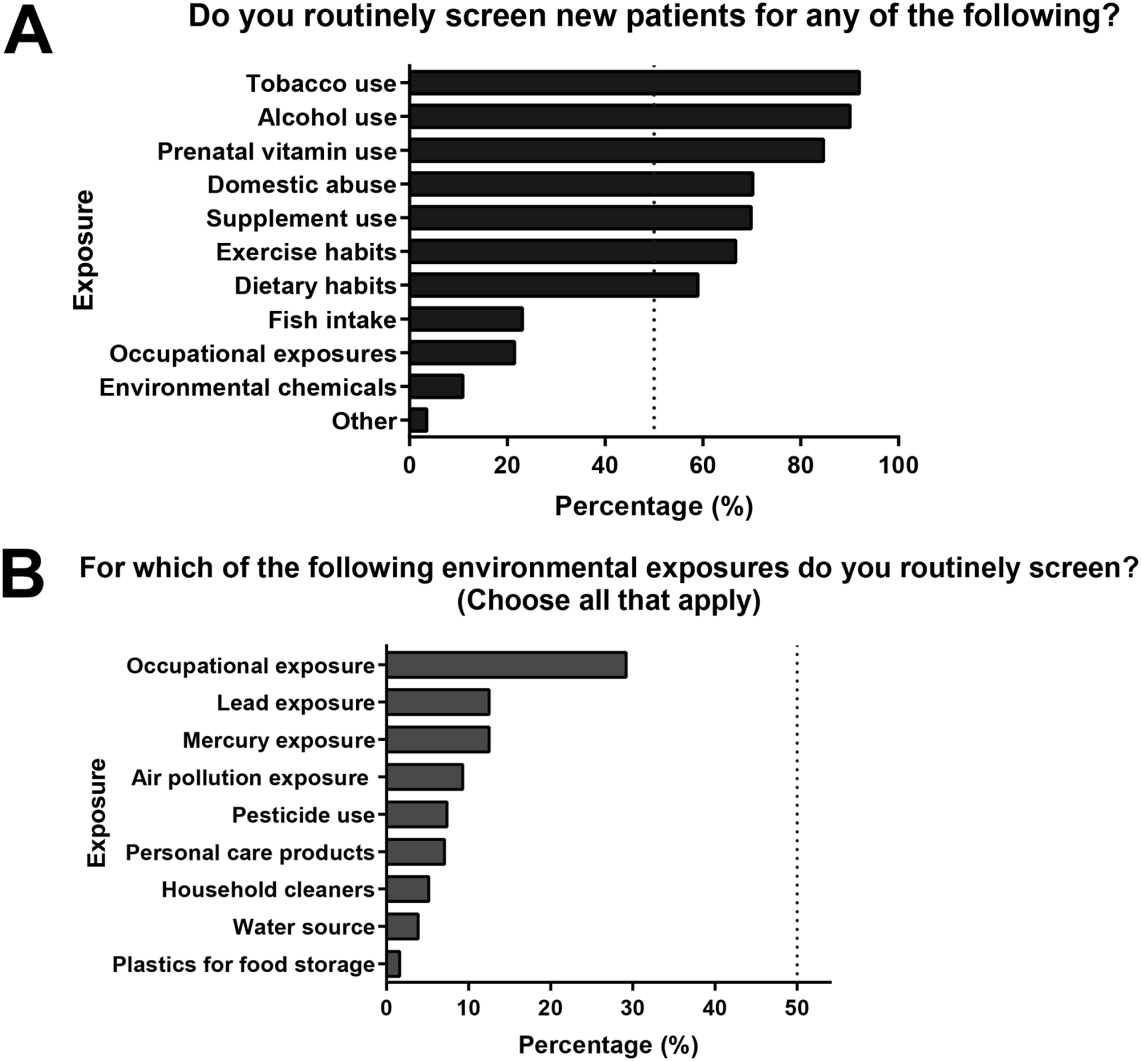
OBGYN screening practices at new patient visits. A.) Responses to the question: do you routinely screen new patients for any of the following (Choose all that apply)? B.) Responses to the question: Which of the following environmental exposures do you routinely screen for? (Choose all that apply).

**Fig 3:**
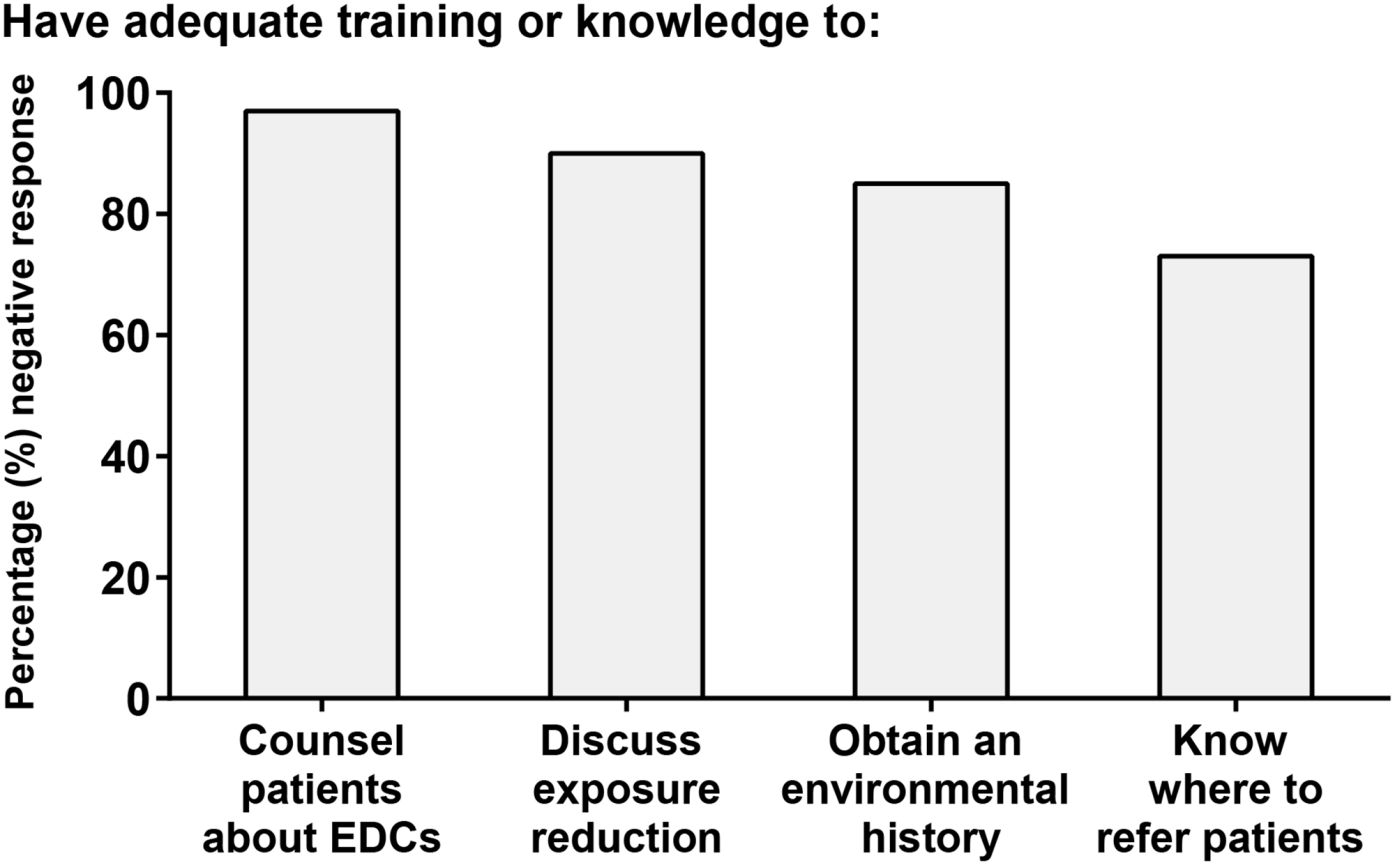
OBGYN comfort level with environmental chemical screening.

More than 50% of respondents screened for the following: prenatal vitamin use, supplement use, alcohol use, tobacco use, domestic violence, dietary, and exercise habits (Fig 2). Fewer than 30% of providers screened for any environmental and occupational exposures (Fig 2). 85% of providers reported that they did not feel comfortable obtaining an environmental history, 58% indicated no regular screening of environmental exposures, and 96% reported that they did not feel they had the knowledge to counsel patients about any associations between environmental chemicals and adverse health. The majority of respondents reported that they did not known how to reduce exposures (90%) and did not know where to refer a patient for more information about environmental chemicals (73%, Fig 3).

We found that respondent gender and practice location (urban/suburban vs. rural) were not significantly associated with differences in environmental literacy or screening practices. A greater frequency of screening for environmental exposures was associated with more than 4 years in practice and not being affiliated with a residency program (Table 2). Residents, as opposed to fully trained OB/GYN generalists and specialists, were the least likely to screen for environmental exposures and least likely to know where to refer a patient for more information (Table 3). Residents were also the least likely to have read the ACOG environmental committee opinion.

**Table 2:** Environmental literacy and screening practices based on years from and affiliation with residency program.

Characteristic	Value	Time since residency Number (% of total)		*p*	Affiliation Number (% of total)		*p*
		<4 years n = 125	4+ years n = 187		Yes n = 170	No n = 135	
**Read ACOG opinions/bulletins**		117(97)	170(91)	0.096	160(94)	127(94)	0.99
**Do you routinely screen new patients for any of the following?** **(Choose all that apply)**	Prenatal vitamin use	103(82)	161(86)	0.424	139(82)	123(91)	0.021
	Supplement use	79(63)	139(74)	0.044	110(65)	107(79)	0.007
	Tobacco use	113(90)	174(93)	0.524	158(93)	127(94)	0.817
	Alcohol use	109(87)	172(92)	0.18	154(91)	125(93)	0.546
	Exercise habits	74(59)	134(72)	0.027	106(62)	101(75)	0.026
	Dietary habits	65(52)	119(64)	0.046	88(52)	95(70)	0.001
	Domestic abuse	87(70)	132(71)	0.9	123(72)	94(70)	0.613
	Fish intake	17(14)	55(29)	0.001	24(14)	47(35)	< .001
	Occupational exposures	17(14)	50(27)	0.007	28(16)	39(29)	0.012
	Environmental chemicals	9(7)	25(13)	0.097	15(9)	19(14)	0.199
**Which of the following environmental exposures do you routinely ask your patients about?** **(Choose all that apply)**	Lead exposure	10(8)	29(16)	0.055	18(11)	21(16)	0.228
	Mercury exposure	8(6)	31(17)	0.008	13(8)	26(19)	0.003
	Pesticide use	6(5)	17(9)	0.188	7(4)	16(12)	0.015
	Occupational exposure	29(23)	62(33)	0.075	35(21)	56(41)	< .001
	Air pollution exposure	9(7)	20(11)	0.327	12(7)	17(13)	0.118
	Plastics for food storage	2(2)	3(2)	.99	3(2)	2(2)	0.99
	Water source	5(4)	7(4)	.99	5(3)	7(5)	0.381
	Personal care products	6(5)	16(9)	0.261	8(5)	14(10)	0.074
	Household cleaners	5(4)	11(6)	0.603	3(2)	13(10)	0.003
**Knowledge to counsel environmental chemicals/adverse health**		2(2)	7(4)	0.330	0 (0.00)	9(7)	< .001
**Training to discuss with a patient how to reduce exposure**		13(12)	16(9)	0. 549	9(6)	20(16)	0.005
**Know where to refer a patient**		24(21)	55(31)	0.079	41(26)	37(29)	0.593
**Comfort obtaining environmental history?**		19(17)	24(14)	0.498	20(13)	23(18)	0.186
**Read Exposure to Toxic Envr. Agents (ACOG/ASRM) or FIGO**		9(8)	27(15)	0. 071	17(11)	19(15)	0.285

Values reported are frequency and column percentage. N = 7 providers not responding to residency affiliation question omitted from Affiliation comparison in Table 2.

**Table 3:** Environmental literacy and screening practices based on respondents’ specialty.

Characteristic	Value	OB/GYN n = 195 Number (% of total)	Specialist n = 72 Number (% of total)	Resident n = 37 Number (% of total)	p
**Read ACOG opinions/bulletins**		181(93)	66(92)	37(100)	0.237
**Do you routinely screen new patients for any of the following?** **(Choose all that apply)**	Prenatal vitamin use	177(91)	52(72)	33(89)	< .001
	Supplement use	146(75)	49(68)	21(57)	0.069
	Tobacco use	183(94)	66(92)	36(97)	0.525
	Alcohol use	179(92)	65(90)	35(95)	0.746
	Exercise habits	139(71)	47(65)	21(57)	0.190
	Dietary habits	129(66)	37(51)	17(46)	0.015
	Domestic abuse	138(71)	48(67)	31(84)	0.168
	Fish intake	59(30)	12(17)	1(3)	< .001
	Occupational exposures	45(23)	19(26)	2(5)	0.029
	Environmental chemicals	17(9)	13(18)	3(8)	0.086
	Other	9(5)	1(1)	1(3)	0.485
**Which of the following environmental exposures do you routinely ask your patients about?** **(Choose all that apply)**	Lead exposure	27(14)	9(13)	3(8)	0.645
	Mercury exposure	32(16)	6(8)	1(3)	0.030
	Pesticide use	15(8)	8(11)	0(0)	0.120
	Occupational exposure	62(32)	24(33)	5(14)	0.064
	Air pollution exposure	23(12)	5(7)	1(3)	0.152
	Plastics for food storage	1(1)	2(3)	2(5)	0.044
	Water source	9(5)	3(4)	0(0)	0.516
	Personal care products	15(8)	6(8)	0(0)	0.202
	Household cleaners	12(6)	4(6)	0(0)	0.343
**Knowledge to counsel environmental chemicals/adverse health**		7(4)	2(3)	0(0)	0.557
**Training to discuss with a patient how to reduce exposure**		22(12)	5(7)	2(6)	0.356
**Know where to refer a patient**		55(30)	22(32)	2(6)	0.007
**Comfort obtaining environmental history?**		29(16)	11(16)	3(9)	0.552
**Read Exposure to Toxic Environmental Agents (ACOG/ASRM) or FIGO Statement**		21(12)	14(20)	1(3)	0.029

Values reported are frequency and column percentage. N = 8 providers not responding to questions about Practice Type omitted from Table 3.

The majority of our subjects (93.5%) reported routinely reading practice bulletins and committee opinions through ACOG. However, only 12% of respondents reported that they had read the Exposure to Toxic Environmental Agents ACOG Committee opinion. Most demographic characteristics did not differ significantly between respondents who had read the specific committee opinion compared to those that had not. There was one exception: Reproductive endocrinologists and infertility physicians were more likely to have read this committee opinion (22 vs 3%, p<0.001). Respondents who read the environmental committee opinion were more likely to screen patients for supplement use, occupational exposures, and environmental chemicals compared to subjects who had not read this document (Table 4). Additionally, respondents who read the environmental committee opinion were more likely to screen new patients for the following environmental exposures compared to OBGYNs that had not read the committee opinion: lead exposure, pesticide use, occupational exposure, plastic use for food storage, and household cleaners (Table 4). Finally, OBGYNs who read the committee opinion were also more likely to report knowledge of how to counsel patients regarding the adverse health outcomes associated with environmental chemicals as well as familiarity with specific resources to where to refer a patient for more information (Table 4).

**Table 4:** Environmental literacy and screening practices by whether subjects had read the environmental committee opinion.

Characteristic	Value	Yes n = 36 Number (% of total)	No n = 254 Number (% of total)	p
**Do you routinely screen new patients for any of the following?** **(Choose all that apply)**	Prenatal vitamin use	35(97)	229(90)	0.222
	Supplement use	36(100)	182(72)	< .001
	Tobacco use	36(100)	251(99)	.99
	Alcohol use	36(100)	245(96)	0.385
	Exercise habits	28(78)	180(71)	0.436
	Dietary habits	27(75)	157(62)	0.142
	Domestic abuse	27(75)	192(76)	.99
	Fish intake	12(33)	60(24)	0.219
	Occupational exposures	14(39)	53(21)	0.021
	Environmental chemicals	10(28)	24(10)	0.004
	Other	3(8)	8(3)	0.144
**Which of the following environmental exposures do you routinely ask your patients about?** **(Choose all that apply)**	Lead exposure	10(28)	29(11)	0.012
	Mercury exposure	8(22)	31(12)	0.116
	Pesticide use	8(22)	15(6)	0.003
	Occupational exposure	18(50)	73(29)	0.013
	Air pollution exposure	5(14)	24(10)	0.552
	Plastics for food storage	3(8)	2(1)	0.015
	Water source	2(6)	10(4)	0.65
	Personal care products	5(14)	17(7)	0.168
	Household cleaners	5(14)	11(4)	0.035
**Knowledge to counsel environmental chemicals/adverse health**		5(14)	4(2)	0.002
**Training to discuss with a patient how to reduce exposure**		4(11)	25(10)	0.99
**Know where to refer a patient**		16(44)	63(25)	0.017
**Comfort obtaining environmental history?**		8(23)	35(14)	0.16
**Environmental exposure survey?**		7(19)	37(15)	0.606

Values reported are frequency and column percentage. N = 22 providers not responding to *read exposure to exposure to toxic environmental agents* question omitted

In sensitivity analysis, when an adaptive step-up Bonferroni adjustment was applied, p-values initially reported ≤0.001 remained statistically significant.

## Discussion

In this cross-sectional survey of practicing US OB/GYNs, the majority of respondents did not screen for environmental or occupational exposures. We identified the following key areas for improved screening: fish intake, occupational exposures, lead exposures, mercury exposures, environmental chemicals, air pollution, pesticide use, personal care products, household cleaners, water, source, and use of plastics for food storage. Reading the ACOG, ASRM, or FIGO environmental exposure committee opinions [6–8] was associated with higher rate of screening for these items. Further, reported familiarity with committee opinions was associated with greater self-reported knowledge to counsel patients regarding the adverse health outcomes associated with environmental chemicals as well as knowledge of where to refer a patient.

We also report that more than 4 years in practice and not being affiliated with a residency program were associated with a greater frequency of screening for environmental exposures. Similarly, we found that residents, compared to generalists and specialists, were the least likely to screen for environmental exposures and were the least likely to know where to refer a patient for more information. Residents were also the least likely to have read the environmental committee opinion. This unexpected finding may be due to the fact that residents maybe the least likely in our profession to have the ability to ask additional questions at initial visits. We could speculate that because of their closer relationships with their patients and their ability to customize patient questionnaires, OBGYNs in community practice may be more likely to screen for these exposures.

To our knowledge, only one other group has evaluated OBGYN screening for environmental exposures several years ago [9]. Our survey, although with a smaller cohort of respondents, had a similar response rate. In the study by Stotland et al (2012), OBGYNs strongly agreed that conducting an environmental health history would identify exposures and help prevent exposures to environmental threats [9]. Additionally, this group identified that OBGYNs believed that assessing environmental exposures through history taking as well as the role of environmental exposures during pregnancy was of great importance. Contrary to the existing literature, we provide suggestions as to specific environmental exposures to target for improvement in environmental health screening as well as by identifying data to support the recommendation for OBGYNs to use the environmental committee opinion as an education resource.

There is a potential for bias in our survey given our relatively low response rate, which could skew the results to reflect a greater concern and care for environmental exposures than exists in the general population of practicing OBGYNs; this would however strengthen our main conclusion that screening is not prevalent in routine practice. A potential limitation of our study is the preponderance of female gender among our respondents. This was likely secondary to recruitment from a female-predominant social media group. Although OBGYN, in general, has become a female-dominant profession, it is important to use caution extrapolating our results to the broader population of practicing OBGYNs. While the number of providers in rural settings is low in this sample (n = 16), the percentage (5%) is similar to the documented percentage practicing in rural settings (6%); caution should be exercised in interpreting null results for comparisons with this group [11].

Another potential limitation of our study is our use of a generated survey instead of a validated instrument. However, no validated surveys exist for examining physician screening in OBGYNs and we encourage further efforts to create standardized survey instruments for assessing progress in screening for environmental exposures. Of 101 comparisons reported, 36 were significantly different using the cutoff of p<0.05; when adjusted for multiple comparisons 8 remained significant. Some differences could have been observed due to chance with others reflective of true differences in the underlying populations parameters we seek to estimate.

In conclusion, we observed a low frequency of comfort with counseling patients about EDCs, discussing exposure reduction, obtaining an environmental history, and knowing where to refer patients. Although physicians interviewed did not full discuss environmental exposures with their patients, they did discuss other important considerations, such as prenatal vitamin use and lead exposure. This suggests that there is potential to add to this list, which would help improve education on environmental exposures. While reading of and familiarity with committee opinions could lead to more frequent screening the observed association might not be causal. Nevertheless, we suggest that improving physician comfort through training and knowledge could potentially increase screening, and incorporating committee opinions into journal club reading could improve comfort by providing a forum for an evidence-based discussion among peers, and potentially increase screening. Improving physician comfort through training and knowledge could potentially increase screening. Incorporating committee opinions into journal club reading could improve comfort by providing a forum for an evidence-based discussion among peers, and potentially increase screening. Ultimately, universal screening recommendations, the language to be used in such a screening, and referrals or actions physicians should consider discussing with patients should be developed for widespread use.

A secondary goal of this project was to increase awareness of the importance of the environment on women’s health and, ultimately, to encourage more providers to consider screening for this exposure. We encourage the publication of environmental health studies in reproductive health, general OBGYN, and primary care journals. In particular, we encourage the editors of these journals to more carefully consider environmental health publications in order to augment awareness of these issues.

Our study demonstrates that the majority of physician do not routinely screen for known environmental toxins. Reading the published environmental agents committee opinion could lead to more frequent screening. Continuing medical education and tools for communicating risks to patients are needed in order to take advantage of the clinician’s opportunity to identify and prevent environmental exposures that threaten reproductive health. We commend the College’s efforts to expand this outreach through their new online screening tool, which will be made available to patients and physicians and includes easy to use resources.

## Supporting information

S1 Table Survey distributed to participants. (DOCX)
