# Supplementary material for: OBGYN screening for environmental exposures: A call for action
Source: PLoS One. 2018 May 16;13(5):e0195375. doi: 10.1371/journal.pone.0195375 (PMC5955561; doi:10.1371/journal.pone.0195375)
Supplement: S1 Table — (DOCX) [file pone.0195375.s001.docx]

**Study Title: OBGYN Screening for Environmental Exposures (OSEE)**

**Principal Investigator: Natalia Grindler, MD**

**COMIRB No: *******

**Version Date: 08-Nov-2016**

You are being asked to be in this research study because you are an OB/GYN provider. To participate, you will complete the following survey, which will take approximately 5 minutes.

This study is designed to learn more about screening for environmental exposures.

There are few risks of participating in the study. The primary risk is loss of confidentiality. Every effort will be made to protect your privacy. All data will be stored in a secure database, and your name or email will never be attached to your survey response.

You have a choice about being in this study. You do not have to be in this study if you do not want to be.

If you have questions, you can call Natalia Grindler, MD at 303-724-8089. You can call and ask questions at any time.

You may have questions about your rights as someone in this study. If you have questions, you can call the COMIRB (the responsible Institutional Review Board). Their number is (303) 724-1055.

By completing this survey, you are agreeing to participate in this research study.

1. Gender
   1. Male
   2. Female
2. How old are you?
3. Which best describes your current level of training?
   1. In training, not board eligible yet
   2. Board eligible
   3. Board certified in general OBGYN
   4. Board certified in sub-specialty
4. How many years ago did you complete training?
5. What state do you practice in?
6. Where is your practice located?
   1. Urban
   2. Suburban
   3. Rural
   4. Other- please describe
7. Which best describes your practice (choose all that apply):
   1. General OBGYN
   2. Gynecology alone
   3. Obstetrics alone
   4. Maternal Fetal Medicine
   5. Family Planning
   6. Gynecology Oncology
   7. Minimally Invasive Surgery
   8. Urogynecology
   9. Reproductive Endocrinology & Infertility
   10. In training (fellow/resident)
   11. Other- please describe
8. What type of practice are you in?
   1. University-based academic practice
   2. Community-based academic practice
   3. Private practice
   4. Other- please specify
9. Is there a residency training program affiliated with your practice?
   1. Yes
   2. No
10. Do you routinely try to read the Committee Opinions and Practice Bulletins through ACOG?
    1. Yes
    2. No
11. Do you routinely try to read the Committee Opinions and Practice Bulletins through your specialty (i.e. ASRM, FIGO, etc)?
    1. Yes
    2. No
    3. N/A
12. Do you routinely screen new patients for any of the following? (Select as many as applicable):
    1. Use of prenatal vitamin
    2. Supplement use (other vitamins, herbs, natural supplements, alternative medicine)
    3. Tobacco use
    4. Alcohol use
    5. Exercise habits
    6. Dietary habits
    7. Domestic abuse
    8. Fish intake (type of fish, frequency of fish consumption, etc)
    9. Occupational exposures (Metals, chemicals, solvents or fumes, loud noise, radiation, etc)
    10. Environmental chemicals (plastic use, pesticide use, canned food use, use of fragrant personal care products, etc)
    11. Other- please specify
13. Does your clinic have a routine survey administered to new patients to identify environmental exposures?
    1. Yes
    2. No
    3. Other- please specify
14. Do you feel you have adequate training to obtain an environmental history on patients? For example, would you be able to ask the appropriate questions to exclude dangerous occupational exposures?
    1. Yes
    2. No
15. Which of the following environmental exposures do you routinely ask your patients about? Choose all that apply
    1. Lead exposure
    2. Mercury exposure
    3. Pesticide use (including insecticides, herbicides, rodenticides)
    4. Occupational exposure (including exposure to metals, solvents, chemicals, radiation, fumes)
    5. Air pollution exposure (i.e living near industrial site, second hand smoke exposure)
    6. Use of plastics for food storage
    7. Water source (i.e. well water)
    8. Exposure to chemicals in personal care products
    9. Exposure to chemicals in household cleaners
16. Do you have adequate knowledge to counsel patients on the specific adverse health outcomes linked to environmental chemicals?
    1. Yes
    2. No
17. Do you believe that you have enough training to discuss with a patient how to reduce exposure to environmental chemicals?
    1. Yes
    2. No
18. Would you know where to refer a patient if they had specific questions about an environmental exposure?
    1. Yes
    2. No
19. Have you read the “Exposure to Toxic Environmental Agents” joint committee opinion (ACOG and ASRM) and/or “Opinion on Reproductive Health Impacts of Exposure to Toxic Environmental Chemicals” (FIGO)?
    1. Yes
    2. No
20. What would help you screen and counsel patients regarding their environmental exposures?
    1. Standardized new patient intake form
    2. Incorporation of information as part of my maintenance of certification
    3. Other- please specify
21. Do you have any additional comments/suggestions?
